# Dissecting the human microbiome with single-cell genomics

**DOI:** 10.1186/s13073-017-0448-7

**Published:** 2017-06-14

**Authors:** Andrew C. Tolonen, Ramnik J. Xavier

**Affiliations:** 1 Broad Institute of MIT and Harvard University, Cambridge, MA 02142 USA; 2 Center for Computational and Integrative Biology, Massachusetts General Hospital and Harvard Medical School, Boston, MA 02114 USA; 3 Gastrointestinal Unit and Center for the Study of Inflammatory Bowel Disease, Massachusetts General Hospital and Harvard Medical School, Boston, MA 02114 USA; 4 Center for Microbiome Informatics and Therapeutics, MIT, Cambridge, MA 02139 USA

## Abstract

Recent advances in genome sequencing of single microbial cells enable the assignment of functional roles to members of the human microbiome that cannot currently be cultured. This approach can reveal the genomic basis of phenotypic variation between closely related strains and can be applied to the targeted study of immunogenic bacteria in disease.

## The human microbiome at the cellular level

The human body is inhabited by a complex collection of microorganisms constituting the human microbiome, which is increasingly recognized as having important roles in human health and disease. Many members of the human microbiome belong to phyla from which no isolates have been cultured owing to their unknown growth requirements, resulting in the widespread application of cultivation-independent methods to characterize the composition and function of the microbiome. For example, the Human Microbiome Project (HMP) is cataloging the healthy human microbiome at multiple body sites by using 16S ribosomal and metagenomic sequencing, providing a reference for future sequencing efforts and prioritizing microbes for study based on their potential importance to human health. Much has been learned about the composition of the microbiome by ribosomal sequencing to resolve taxonomy and by metagenomics to assess the collective gene pool. However, these methods are generally unable to reconstruct how DNA is compartmentalized into cells, which is needed to understand population structure with the cell as the basic unit. Now, single-cell genomics of microbial cells has become possible in recent years and offers a solution to this limitation. Furthermore, it can define the metabolic features and pathogenic potential of specific bacterial cells and can indicate whether they contain phage and plasmids that facilitate horizontal transfer of genes for clinically relevant traits, such as antibiotic resistance.

## Advances and challenges in microbial single-cell sequencing

**Fig. 1:**
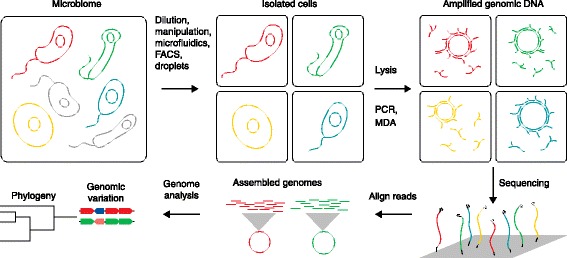
Overview of steps for single-cell sequencing of microbial genomes. Experimental steps include (*upper half*) isolation and lysis of single cells with subsequent amplification of their genomes, followed by (*lower half*) high-throughput sequencing, genome assembly and genome analysis. *FACS* fluorescence-activated cell sorting, *MDA* multiple displacement amplification, *PCR* polymerase chain reaction

Single-cell sequencing of microbial genomes entails technical challenges relating to the various steps of the required workflow: isolation of individual cells, whole-genome amplification, DNA sequencing, and sequence analysis (Fig. 1). Several approaches have been developed to isolate single cells by using either serial dilution, microfluidics, flow cytometry, micromanipulation, or encapsulation in droplets [1]. These methods permit targeted isolation of a cell from mixed populations in liquid medium, but isolating microbial cells from primary samples such as swabs and biopsies remains challenging, especially from solid tissues requiring homogenization. Once the cell has been isolated, the cell envelope is broken using a procedure that is rigorous enough to rupture recalcitrant taxa, but delicate enough to limit chromosomal break-points that will not be covered in the final sequence.

Genomic DNA must then be amplified into a library containing many copies of each locus for genome sequencing. The gold standard for genome amplification is multiple displacement amplification (MDA) using a strand-displacing polymerase such as the proof-reading Phi29 polymerase with random, phosphorothioate-modified primers to synthesize long, overlapping products. The single-stranded products of MDA are substrates for further synthesis, which increases amplification but also creates problems when they anneal and prime synthesis elsewhere in the genome. This leads to the formation of 'chimeric DNA' that links non-adjacent template sequences. Initially, DNA chimeras were present in 20% of sequences and impeded assembly [2], but problems with chimeras have since been minimized with improved protocols and increased sequencing depth [3].

Next, the amplified DNA is sequenced on a high-throughput platform and the reads are then assembled. Conventional genome-assembly algorithms often have problems with single-cell data because they assume that chimeras are rare and genome coverage is Poisson distributed. Biochemical normalization procedures [3] and assembly algorithms such as Velvet-SC and SPAdes have been developed to control for these biases [1].

In addition to MDA-based amplification of single genomes, alternative methods have emerged to increase sequencing depth and genome assembly from microbiome samples. Fusion PCR on individual cells encapsulated in polyacrylamide beads facilitates deep sequencing of the phylogenetic distribution of target genes in a mixed population [4]. TruSeq synthetic long-read sequencing is another high-throughput approach to reveal intraspecific haplotype diversity and rare species in the gut microbiome [5]. Genome assembly, especially of rare species, can be improved with 'mini-metagenomics' by flow-sorting cells into pools of a few hundred cells that are together subjected to MDA [6]. Gel microdroplet (GMD) cultivation [7] is yet another method in which single cells are encapsulated in agar droplets and grown to a population of hundreds of cells before MDA. GMD simplifies genome assembly, but it can introduce sampling bias because the cells must be able to grow and divide in the agar beads.

These technology advances to perform single-cell sequencing of bacteria are enabling new investigations into the roles of specific taxa of the human microbiome in health and disease.

## The promise of targeted single-cell sequencing of the human microbiome

Single-cell genomics of the human microbiome has already led to the discovery of bacteria with novel metabolic features, and even an alternative genetic code [8]. Owing to the diversity of taxa in the microbiome, a method such as 16S sequencing after MDA or antibody-based immunomagnetic separation must be used to prioritize individual cells from mixed samples for genome sequencing. For example, the first whole genomes produced from clinical samples were of *Chlamydia trachomatis* cells isolated from swabs by capture on magnetic beads using a mouse immunoglobulin G (IgG) primary antibody that specifically binds *C. trachomatis* lipopolysaccharide [9]. Antibodies could be generally applied to isolate cells of interest for genome sequencing based on cell-surface markers.

Microbes can also be selected for single-cell genome sequencing based upon their recognition by the host immune system. Immunoglobulin A (IgA), the main antibody isotype produced at mucosal surfaces, binds pathogens in the intestinal lumen. Cell sorting using a fluorescent anti-IgA antibody followed by 16S rDNA sequencing selectively identifies microbial taxa that induce inflammation and drive intestinal disease [10]. Similarly, anti-IgG-based isolation of bacteria could be applied to study the genomes of bacterial cells inducing a systemic immune response. In particular, the IgG response to gut bacteria under homeostatic conditions protects against systemic infections such as sepsis, and Crohn’s disease patients show elevated IgG coating of gut bacteria [11] likely resulting from impaired mucosal barrier function. Selecting cells for single-cell genome sequencing based on immunoglobulin coating could identify the basis of immunogenic differences between, and perhaps within, bacterial species in the gut microbiome.

## Conclusions and future directions

These emerging approaches in single-cell genomics will identify fine-scale genomic variation between strains to help elucidate the mechanisms by which the human microbiome interacts with its host to influence health and disease. Analysis of individual genomes from the human microbiome can also be broadly applied in fields such as epidemiology to trace the emergence of pathogens and drug-resistant strains.

## Abbreviations

16S: a subunit of the prokaryotic ribosome
FACS: Fluorescence-activated cell sorting
GMD: Gel microdroplet
IgA/IgG: immunoglobulin A/G
MDA: Multiple displacement amplification
